# Supplementary material for: Food insecurity is associated with decreased quality of life in patients with chronic Chagas disease
Source: PLoS One. 2025 Jul 16;20(7):e0328466. doi: 10.1371/journal.pone.0328466 (PMC12266387; doi:10.1371/journal.pone.0328466)
Supplement: S1 Table — (DOCX) [file pone.0328466.s001.docx]

**Table 1.** Clinical and demographic characteristics of participants (n= 359).

| Variables | Overall |
| --- | --- |
|  | (100%; n=359) |
| **Age (Years) (Median; IQR 25% - 75%)** | 62 (54 – 69) |
| **Sex (%; n)** |  |
| Women | 56% (201) |
| **Race (%; n)^†^** |  |
| Non-white (*vs* white) | 77.4% (278) |
| **Schooling (%; n)** |  |
| <9 years | 67.7% (243) |
| 9-12 years | 18.4% (66) |
| >12 years | 13.9% (50) |
| **Region of origin (%; n)** |  |
| North | 0.8% (3) |
| Northeast | 68.5% (246) |
| Southeast | 26.5% (95) |
| South | 2.2% (8) |
| Central west | 1.4% (5) |
| Other countries^‡^ | 0.6 (2) |
| **Nutritional status (%; n)** |  |
| Underweight | 2.2% (8) |
| Eutrophic | 30.6% (110) |
| Overweight | 41.5 (149) |
| Obesity | 25.6% (92) |
| **Macronutrients intake (Grams) (Median; IQR 25% - 75%)** |  |
| Carbohydrate | 179.1 (137.7 - 241.4) |
| Lipid | 34.9 (25.2 - 49) |
| Protein | 62.7 (45.8 - 82.1) |
| Fiber | 16.5 (11.4 - 23.4) |
| **Caloric consumption (Kcal) (Median; IQR 25% - 75%)** | 1209.5 (869.1 - 1614.7) |
| **Hypertension (%; n)** | 67.1% (241) |
| **Diabetes Mellitus (%; n)** | 21.7% (78) |
| **Dyslipidemia (%; n)** | 53.8% (193) |
| **Chagas disease forms^**^** |  |
| Indeterminate form (%; n) | 27% (97) |
| Cardiac form without heart failure (%; n) | 54.3% (195) |
| Cardiac form with heart failure (%; n) | 15.3% (55) |
| Digestive form (%; n) | 15.9% (57) |
| **Per capita family income (R$) (Median; IQR 25% - 75%)** | 750 (440 – 1000) |
| **BMI (Kg/m^2^) (Median; IQR 25% - 75%)** | 26.8 (23.7 - 30.1) |
| **Weight (Kg) (Median; IQR 25% - 75%)** | 67.5 (59 – 78.6) |
| **Height (m) (Median; IQR 25% - 75%)** | 1.5 (1.5 - 1.6) |
| **Waist circumference (cm) (Median; IQR 25% - 75%)** | 89.8 (81.7 - 98.3) |
| **Total cholesterol (mg/dl) (Median; IQR 25% - 75%) n=353** | 184 (161 – 206) |
| **Triglycerides (mg/dl) (Median; IQR 25% - 75%) n=352** | 102.5 (69.5 - 144) |
| **HDL (mg/dl) (Median; IQR 25% - 75%) n=303** | 48 (40 – 58) |
| **LDL (mg/dl) (Median; IQR 25% - 75%) n=302** | 111 (92 – 132) |
| **VLDL (mg/dl) (Median; IQR 25% - 75%) n=350** | 20 (14 – 28) |
| **Glucose (mg/dl) (Median; IQR 25% - 75%) n=358** | 96 (89 – 106) |
| **Quality of life-related domains (WHOQOL-BREF)** |  |
| **Physical health domain (Median; IQR 25% - 75%)** | 60.7 (46.4 - 71.4) |
| **Psychological domain (Median; IQR 25% - 75%)** | 70.8 (58.3 - 79.2) |
| **Social relationship domain (Median; IQR 25% - 75%)** | 75 (58.3 - 75) |
| **Environment domain (Median; IQR 25% - 75%)** | 56.2 (46.9 - 65.6) |
| **Overall domain (Median; IQR 25% - 75%)** | 62.5 (50 – 75) |
